# SONAR: A High-Throughput Pipeline for Inferring Antibody Ontogenies from Longitudinal Sequencing of B Cell Transcripts

**DOI:** 10.3389/fimmu.2016.00372

**Published:** 2016-09-21

**Authors:** Chaim A. Schramm, Zizhang Sheng, Zhenhai Zhang, John R. Mascola, Peter D. Kwong, Lawrence Shapiro

**Affiliations:** ^1^Department of Biochemistry and Molecular Biophysics, Columbia University, New York, NY, USA; ^2^Department of Systems Biology, Columbia University, New York, NY, USA; ^3^Vaccine Research Center, National Institute of Allergy and Infectious Diseases, National Institutes of Health, Bethesda, MD, USA

**Keywords:** antibody repertoire, antibody lineage, antibody maturation, B cell ontogeny, longitudinal analysis, next-generation sequencing

## Abstract

The rapid advance of massively parallel or next-generation sequencing technologies has made possible the characterization of B cell receptor repertoires in ever greater detail, and these developments have triggered a proliferation of software tools for processing and annotating these data. Of especial interest, however, is the capability to track the development of specific antibody lineages across time, which remains beyond the scope of most current programs. We have previously reported on the use of techniques such as inter- and intradonor analysis and CDR3 tracing to identify transcripts related to an antibody of interest. Here, we present Software for the Ontogenic aNalysis of Antibody Repertoires (SONAR), capable of automating both general repertoire analysis and specialized techniques for investigating specific lineages. SONAR annotates next-generation sequencing data, identifies transcripts in a lineage of interest, and tracks lineage development across multiple time points. SONAR also generates figures, such as identity–divergence plots and longitudinal phylogenetic “birthday” trees, and provides interfaces to other programs such as DNAML and BEAST. SONAR can be downloaded as a ready-to-run Docker image or manually installed on a local machine. In the latter case, it can also be configured to take advantage of a high-performance computing cluster for the most computationally intensive steps, if available. In summary, this software provides a useful new tool for the processing of large next-generation sequencing datasets and the ontogenic analysis of neutralizing antibody lineages. SONAR can be found at https://github.com/scharch/SONAR, and the Docker image can be obtained from https://hub.docker.com/r/scharch/sonar/.

## Introduction

Antibodies, the soluble form of B cell receptors (BCRs), play a critical role in adaptive immunity. Approximately 50 million naive B cells are generated *via* V(D)J recombination in the bone marrow each day. Due to the combinatorial possibilities of recombination and the inclusion of non-templated “N” and “P” nucleotides, each naive B cell generally expresses a unique BCR (1). If a naive B cell encounters an antigen that can be bound by its receptor and is stimulated by a cognate T cell, it will begin proliferating. As B cells proliferate, they express activation-induced cytidine deaminase, which causes the rapid accumulation of somatic hypermutation in the BCR gene (2). Daughter cells descended from the same naive B cell form a B cell lineage. The typical human B cell repertoire has been estimated to contain ~30,000 highly expanded IgM, IgG, and IgA lineages as well as ~5 million low-expansion IgM lineages at any given time (3).

The mutated BCRs expressed by the cells of a B cell lineage are selected for binding to antigen. In this way, the adaptive immune system can produce antibodies capable of binding to and protecting against nearly any invading pathogen. Most effective vaccines work by eliciting neutralizing antibodies (4), and many recombinant antibodies are now being used as therapeutics (5). In addition, B cell dysfunction may result in autoimmune diseases, such as systemic lupus erythematosus (6), and various B cell lymphomas (7, 8), among others. Understanding each of these B cell-related diseases requires knowledge of the properties and dynamics of natural antibody repertoires and how these properties change in response to factors such as age, vaccination, and disease.

A particularly important area of research is the generation and development (ontogeny) of individual B cell lineages and ontogeny-based vaccine design (9). These studies can reveal not only the mechanisms of modulating antibody-affinity maturation and neutralization breadth development (2, 10–12) but also help to find related antibodies that are more suitable for use as therapeutics (13–15). However, several obstacles must be overcome to define the history and maturation of a single lineage. First, out of a total repertoire of millions of antibody lineages (3, 16), even a highly expanded lineage may constitute at most only up to 0.1% of the overall B cell population (16). Thus, careful selection procedures and/or extensive sampling are required in order to gain sufficient representation. The rapid development of next-generation sequencing technology (17–19) has ameliorated the first of these problems. It is now possible to obtain millions of reads quickly and cheaply, making it possible to sample the antibody repertoire at great depth. To help manage and process these data, a wealth of software tools have been introduced, most notably IMGT-vQuest (20), JoinSolver (21, 22), and IgBlast (23), as well as more recent tools such as VDJSeq-Solver (24), ImmunediveRsity (25), IMonitor (26), CloAnalyst (27, 28), and partis (29).

Even with adequate sampling, it can be difficult to determine which antibodies are members of the same B cell lineage, as there will generally be multiple lineages which share the same V and J gene. The recombination region – including 5′ and 3′ excisions, N and P added nucleotides, and (for heavy chains) the choice of D gene – is generally regarded as a definitive signature of membership in a single B cell lineage [e.g., Ref. (3, 25, 30–32)]. However, such signatures can be obscured by sequencing error and somatic hypermutation (12, 33), unless patterns of mutations across the entire variable region are taken into account (34).^1^ The light chains of a lineage are even more difficult to assess, as they do not contain a D gene. A somewhat simpler problem than *de novo* or “unseeded” lineage identification is finding only those transcripts which are in the same lineage as a known “seed” antibody sequence, such as an antibody identified by cell sorting or culture. We have previously reported several methods for addressing this question, including identity–divergence plots (35, 36), inter- and intra donor phylogenetic analysis (11, 12, 35), and CDR3 clustering (12, 35).

Once a group of transcripts in a lineage have been identified, phylogenetic analysis can be used to build a tree showing how the lineage developed and infer the sequence of unobserved ancestral sequences. While a few tools are available for this task (27, 37, 38), they do not distinguish transcripts from different time points or allow direct and explicit analysis of how a lineage evolves over time. Longitudinal information can be extremely important, however, for indicating whether a lineage is static or continuing to mature (12) and providing the ability to trace co-evolution with a viral pathogen (10, 11, 39, 40).

Here, we present the Software for the Ontogenic aNalysis of Antibody Repertoires (SONAR), an integrated pipeline for performing all of these types of analyses in a single environment. SONAR focuses on the analysis of longitudinal data to understand the development of a single antibody lineage over time. Early versions of this pipeline were used to successfully trace the development of broadly neutralizing antibodies against HIV-1 such as CAP256-VRC26 (11, 39, 41) and VRC01 (12); it has now been extensively overhauled for efficiency and readability, and many new features have been added. Here, we release SONAR as open software under the GNU General Public License. SONAR source code is available from GitHub or as a platform-independent Docker image with all required dependencies already installed.

## Materials and Methods

### Computer Hardware and Software Requirements

The SONAR pipeline can be run on any operation system (OS) using the Docker image found at https://hub.docker.com/r/scharch/sonar/. Local installation is available for Unix-based operating systems and requires Python 2.7 with the BioPython package (42); Perl 5 or higher with the BioPerl module (43); R with the ggplot2, grid, and MASS libraries; and BLAST+ (44). For full functionality, the following programs are also required: FASTX-Toolkit,^2^ USEARCH v8 (45), MUSCLE v3.8 (46), DNAML (47), BEAST2 (48), the ete2 Python package (49), and docopt for Python and R.^3^

### License and Distribution

Software for the Ontogenic aNalysis of Antibody Repertoires is made available under the GNU General Public License, version 3. Permission is granted to modify and redistribute SONAR in any fashion so long as the original copyright notice remains intact and any changes are clearly marked. Source code can be downloaded from https://github.com/scharch/SONAR.

### Reference Germline Gene Sequences

Reference human germline gene sequences were downloaded from the IMGT database (release 201631-4, August 4, 2016). Alleles marked by IMGT as “ORF” or “P” are excluded from the default databases; however, files with all IMGT alleles are included, as well.

### Sample Deep-Sequencing Data

The examples shown here make use of previously published 454 data from donor CAP256 (11) and can be downloaded from the NCBI Sequence Reads Archive under accession number SRP034555.

## Results

### Overview of SONAR

To run SONAR locally, download the source code from GitHub and run the setup.sh bash script. This script will ask for the installation paths of needed accessory programs and make this information available to the main SONAR programs. The setup.sh script also allows SONAR to be set up to use a Grid Engine-managed computing cluster, enabling parallel processing of large datasets.

The setup procedure only needs to be run the first time that SONAR is downloaded; updates to the source code can be downloaded without overwriting user-specific data. Alternatively, a ready-to-use Docker image can be obtained from Docker hub and run using the command:

docker run -i -t -v /path/to/local/project:/project scharch/sonar

where <project> is the name of project with data to be analyzed, and the path indicates its location on the local disk.

Because many different sequencing protocols are used to generate antibody repertoire data, SONAR expects transcripts that have already been preprocessed, if necessary. This can include separating different experiments based on barcodes and/or collapsing redundant transcripts using molecular ID tags. SONAR does offer a script to merge paired-end reads from the Illumina MiSeq platform and to remove transcripts with the expected number of errors above a chosen threshold using USEARCH (45), but other forms of quality control must be performed manually before running the SONAR pipeline.

**Figure 1:**
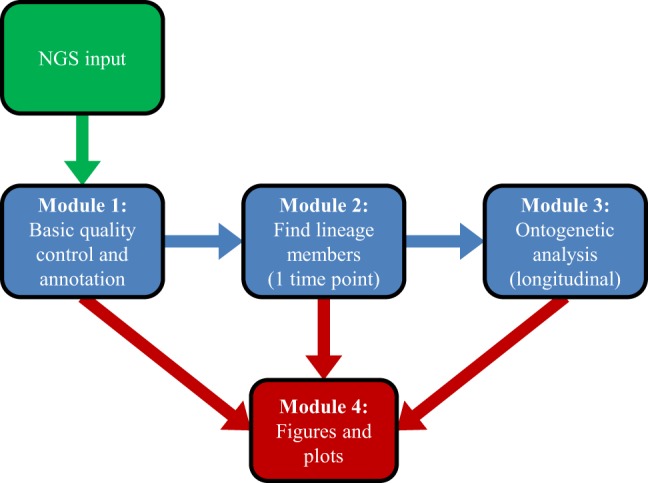
**Overview of SONAR workflow**. Green represents input data, blue indicates analysis steps, and red denotes graphical output.

Software for the Ontogenic aNalysis of Antibody Repertoires proceeds in three conceptual steps (Figure 1). First, it annotates the bulk transcripts using BLAST+ (44), which produces a picture of the overall repertoire sampled by a single experiment. Second, SONAR attempts to classify transcripts into distinct lineages, using either seeded or unseeded techniques. Finally, SONAR combines related transcripts from multiple time points or experiments to conduct an ontogenic analysis.

All SONAR scripts can be called with a -h or -help option to print detailed documentation and usage options at the command line. This documentation will also typically be produced if a script is called with insufficient or incorrectly formatted options.

### Module 1: Annotation

**Figure 2:**
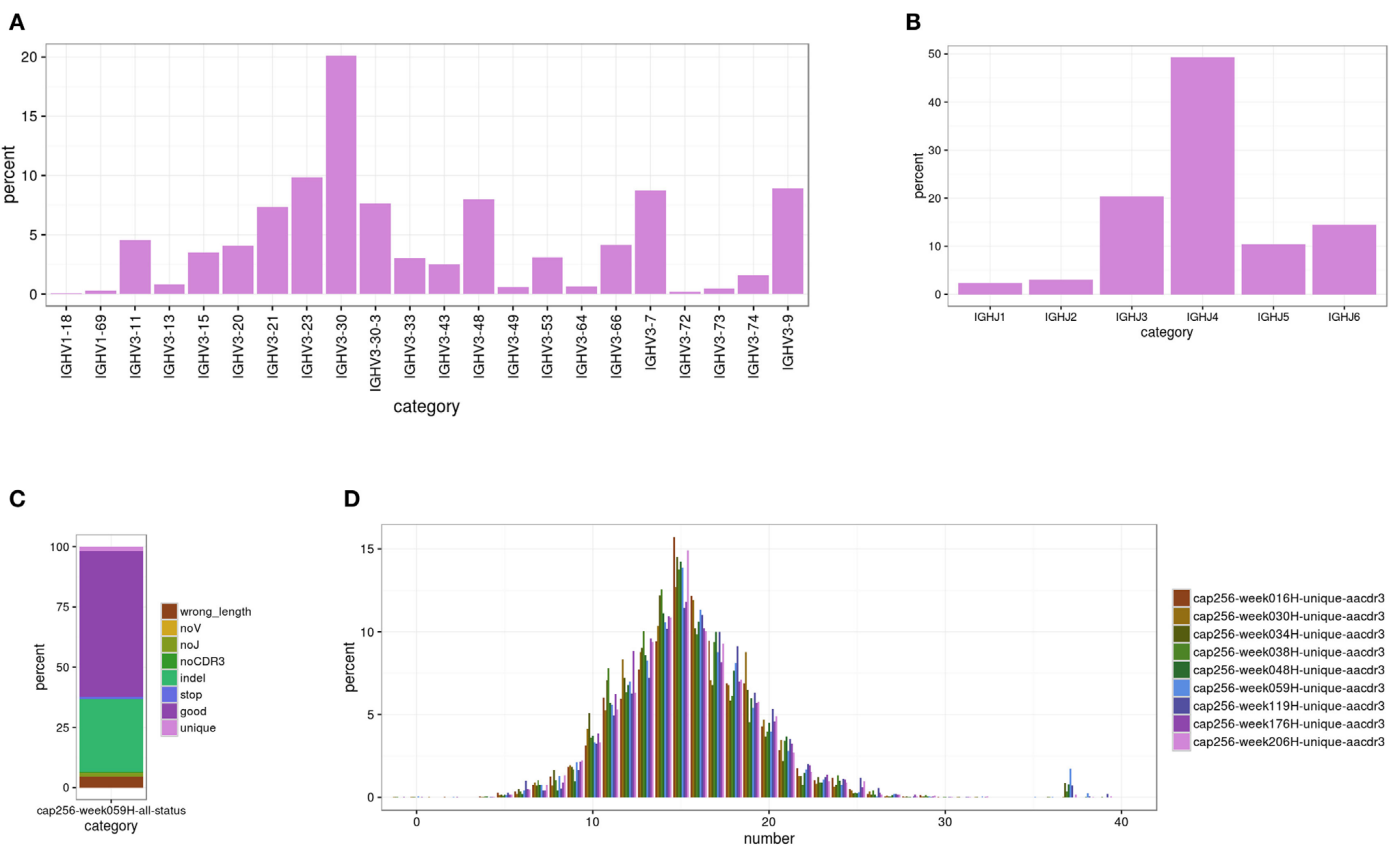
**Sample figures for Module 1 analyses**. **(A)** The V gene usage for donor CAP256 at 59 weeks post-infection (SRA ascension SRX395942). cDNA was amplified with VH3 family-specific primers; the peak for VH3-30 is from the expansion of the CAP256-VRC26 lineage. **(B)** The J gene usage for the same dataset. The CAP256-VRC26 lineage uses JH3. **(C)** The status assigned to each transcript in this dataset by SONAR. Approximately 30% of the transcripts are identified as containing in-dels (light green), which is typical for uncorrected 454 data. Approximately 60% are high-quality transcripts (dark purple), but only ~2% of these (~3% overall) are non-redundant at the 97% threshold (light purple). **(D)** CDR H3 length distribution (in amino acids, IMGT delineation) for all nine donor CAP256 time points. The CAP256-VRC26 lineage can be seen in the peak at 37 amino acids, which first appears at the 34 weeks post-infection time point.

This module characterizes the overall repertoire captured by sequencing. To do so, the germline V(D)J gene of each transcript is assigned using BLAST+ with optimized parameters. Because IgBlast was not available as a stand-alone program that could be run locally when we began building SONAR, we developed separate scripts to find the V and J genes and assign the boundaries of CDR3 using the alignment boundaries output by BLAST. While a blunt tool, such as BLAST, cannot resolve uncertainty in the assignment of the exact allele of a particular germline gene used in recombination (29), SONAR is designed primarily for use with highly mutated neutralizing antibody sequences, for which a definitive assignment is often not possible. SONAR does report the top allele found by BLAST but only uses the gene for all phylogenetic analyses. In addition, the exact alleles carried can vary widely among different donors (50), and this information is typically not available. Similarly, SONAR currently makes no attempt to assign the exact boundaries of recombination, as this information is often obscured for highly mutated antibodies (29). In addition, the IMGT databases included in the distribution contain some alleles with identical sequences but multiple designators (e.g., IGHV3-30*18 and IGHV3-30-5*01 or IGKV1-12*02 and IGKV1D-12*02), which cannot be distinguished by BLAST, and SONAR shares this limitation. The output from this module includes a master table with the disposition of each input transcript and summary statistics for gene usage. This information can be passed to the plotting module to create figures describing the repertoire (Figure 2).

#### 1.0-MiSeq_assembly.pl

This optional script merges paired-end reads from Illumina MiSeq (or HiSeq) and removes reads that cannot be merged or are of low quality. Trimming is done *via* the FastX Toolkit, and merging is done with USEARCH. Prior to merging, reads can be trimmed by a specific number of nucleotides or based on quality scores. Low quality reads can be discarded after merging using the number of expected miscalled bases (as calculated by USEARCH from the quality scores at each position).

#### 1.1-blast_V.py

This script initiates the analysis for each project. The name of the current working folder is used as the project name, which is used as the stem for all output files. New directories are created for working files and processed output. If the work or output directories already exist, the script exits with an error unless the -f (force) flag has been specified. This prevents accidental overwriting of existing data.

By default, all fasta and fastq files in the work directory are processed, but a specific file or files can be stipulated. Reads which are too short or too long to correspond to an antibody variable region are discarded. Input sequences are broken into groups and blasted against a library of germline V genes. Human heavy, kappa, and lambda libraries are included with the source code, but a custom library can be specified using the -lib option. By default, BLAST+ is run locally using one thread; however, multiple threads can be used or the individual blast jobs can be submitted to a cluster if one is present.

#### 1.2-blast_J.py

This script parses the output of BLAST+ from 1.1-blast_V.py to extract the assigned germline V gene and generates new BLAST+ jobs to search for the germline J gene. To improve assignment efficiency, only the portion of the NGS transcript after the 3′ end of the V gene match is scanned; transcripts with no matched V gene are discarded. By default, this script also uses BLAST+ to assign the constant region and D gene for heavy chain transcripts, but this functionality can be disabled to speed up processing time. Outputs from this script are text tables in output/tables with the top V gene hit for each transcript and a summary of how many times each V gene allele is observed in the dataset.

#### 1.3-finalize_assignments.py

This script parses the output of BLAST+ from 1.2-blast_J.py to extract the assigned germline J gene and uses the boundaries of the V and J gene alignment to extract CDR3. Each transcript is also checked for frameshifts and stop codons, and a final status is assigned. Outputs in output/tables include top assignments and summary tables for J genes (plus D genes and constant regions, if applicable). In addition, a master table is generated indicating the source, characteristics, and disposition of each transcript. In output/sequences are files with various subsets of the input sequences, including all transcripts with successful V and J assignments, successful CDR3 extraction, and transcripts with all of the above plus no detected frameshifts or stop codons. Data about the repertoire can be visualized using 4.1-setup_plots.pl (Figure 2).

#### 1.4-dereplicate_sequences.pl

This script uses USEARCH to eliminate redundant transcripts and those below a given sequencing depth threshold. Clustering is also used to account for the introduction of error during PCR and sequencing, eliminating artificial diversity (36).The default identity threshold for clustering is 99%, and only clusters containing at least three transcripts are retained. Both parameters can be adjusted by the user.

### Module 2: Lineage Determination

The process of classifying a set of NGS transcripts into component lineages without any additional information is termed “unseeded lineage assignment.” By contrast, “seeded lineage assignment” uses the sequences of one or more known antibodies as seeds to find all transcripts in the dataset that are from the same lineage, while leaving the remainder of transcripts unclassified. Unseeded lineage assignment is typically accomplished by clustering transcripts based on sequence similarity in CDR3 (3, 25, 30–32), though more sophisticated algorithms have recently been described (34, see footnote text 1). SONAR offers 2.4-cluster_into_groups.py to carry out unseeded lineage assignment, but the suite overall focuses more heavily on seeded lineage assignment, since phylogenetic analysis is carried out on specific lineages. We have previously demonstrated several techniques for effective and efficient seeded lineage assignment, which are included in Module 2 of SONAR (11, 35, 36, 40, 51).

#### 2.1-calculate_id-div.pl

**Figure 3:**
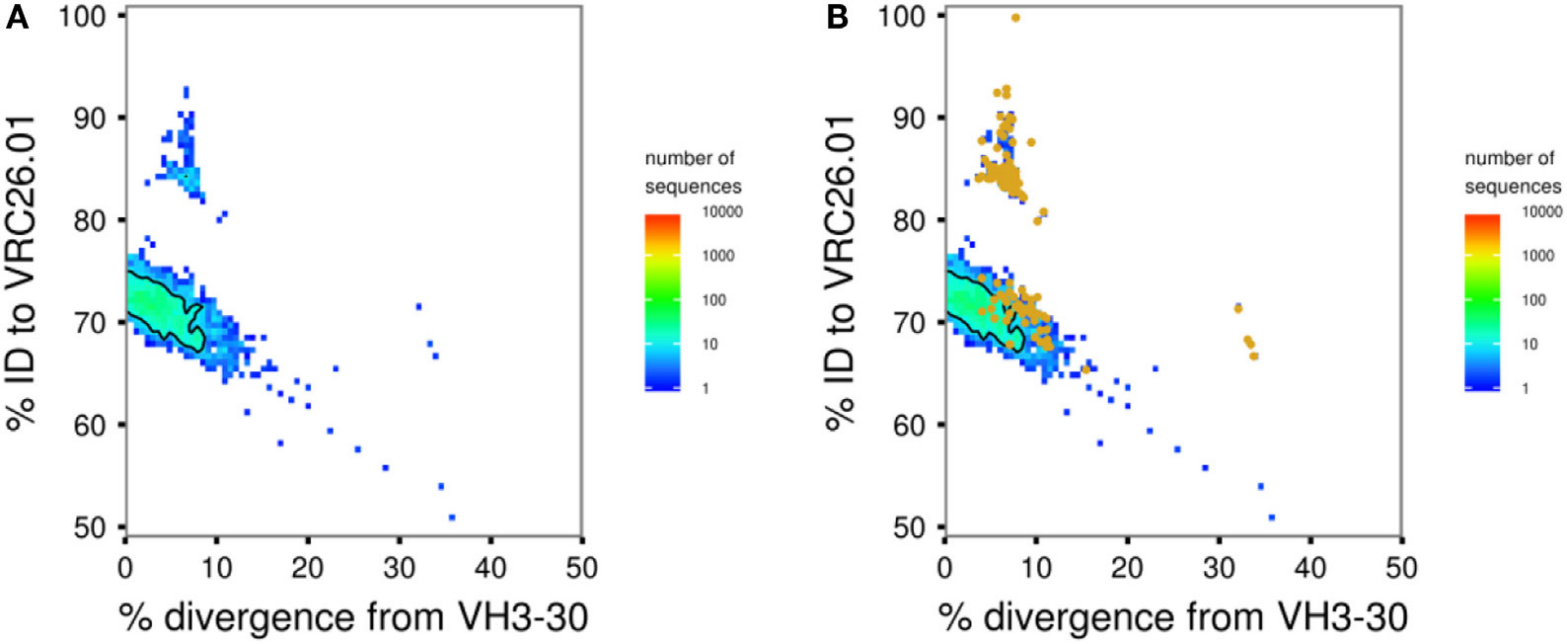
**Sample figures for Module 2 analyses**. **(A)** Identity–divergence plot of transcripts assigned to VH3-30 for donor CAP256 at 59 weeks post-infection. Bulk sequencing data are shown as a heat map with colors as indicated. The CAP256-VRC26 lineage is visible as a distinct island of transcripts at higher identity. **(B)** The same plot with transcripts identified as likely lineage members by intradonor analysis overlaid as orange points. Two thirds of these transcripts are found in the high-identity island; the remaining third in the main body of transcripts at ~70% identity are false positives. This is a typical result, showing why multiple tools for lineage determination are included in SONAR and manual curation is strongly advised.

This script carries out seeded lineage assignment, using Muscle (46) (the default), ClustalO (52), or MAFFT (53) to align each transcript to its assigned germline sequence and to known antibody sequences of interest. Output is a table with the percent identity of each transcript to each of the specified known antibody sequences and its percent divergence from germline V gene. These data can be visualized using 4.3-plot_identity_divergence.R (see below) to identify “islands” of transcripts that are likely to be in the same lineage as an antibody or antibodies of interest (Figure 3A).

#### 2.2-get_island.py

Once an island of transcripts likely to be in the same lineage as the seed antibody has been identified on an identity–divergence plot, this script can be used to extract the transcripts in the island and save them to a new file in output/sequences/nucleotide.

#### 2.3-intradonor_analysis.py

This script offers a second method to perform seeded lineage assignment by using an iterative phylogenetic analysis to find transcripts, which are in the same lineage as set of known antibodies. Transcripts are randomly split into groups and used together with known antibody sequences to build neighbor-joining trees rooted on the germline V gene of the known antibodies. Transcripts in the minimum sub-tree spanning all of the known sequences are passed forward into the next iteration. The algorithm is considered to have converged when 95% of the input sequences in a round are in the minimum sub-tree, and these transcripts are deemed to be in the same lineage as the known antibodies. The algorithm is generally intended to find somatically related antibodies from a single lineage within a single donor. However, in the special case of VRC01 class antibodies (35), we have shown that exogenous VRC01 class heavy chains can be used for “cross-donor” analysis to identify a lineage of VRC01 class antibodies within a new donor (35, 54). For both intradonor and cross-donor analysis, the accuracy and specificity of the algorithm depends on the number of seed sequences used and how closely related they are. Various filtering options are available for the transcripts before starting the analysis, and the tree-building steps of each iteration can be submitted to a high-performance computing cluster, if available. 4.3-plot_identity_divergence.R can be used to overlay the transcripts thus identified as in the same lineage on the visualization of the overall repertoire (Figure 3B).

#### 2.4-cluster_into_groups.py

This script provides both a third technique for seeded lineage assignment and a basic approach for unseeded lineage assignment. Antibody transcripts are first separated into groups based on assigned V and J genes. The transcripts in each group are then clustered based on their CDR3 nucleotide identity using the UCLUST algorithm in USEARCH, and each cluster is identified as a distinct unseeded lineage. Known antibodies of interest can also be included among the transcripts to be clustered, allowing seeded lineage assignment for one or more lineages (12, 35).

### Module 3: Phylogenetic Analysis

Once transcripts in the lineage of the seed antibodies have been identified from one or more cross-sectional samples, the overall phylogenetic structure of an antibody lineage can be examined and the ontogeny of the lineage can be inferred. This includes building and analyzing a phylogenetic tree, inferring intermediates along the maturation pathway of an interested antibody, as well as estimating the evolutionary rate of the lineage over time.

#### 3.1-merge_timepoints.pl

This script collects transcripts in the lineage of the seed antibodies identified at multiple time points using Module 2 and renames them to indicate their temporal origins. A unique label may be specified for each file, such as a sample date or visit code. This script then identifies and collapses transcripts that appear at multiple time points and assigns a “birthday” based on the first observation.

#### 3.2-run_DNAML.py

**Figure 4:**
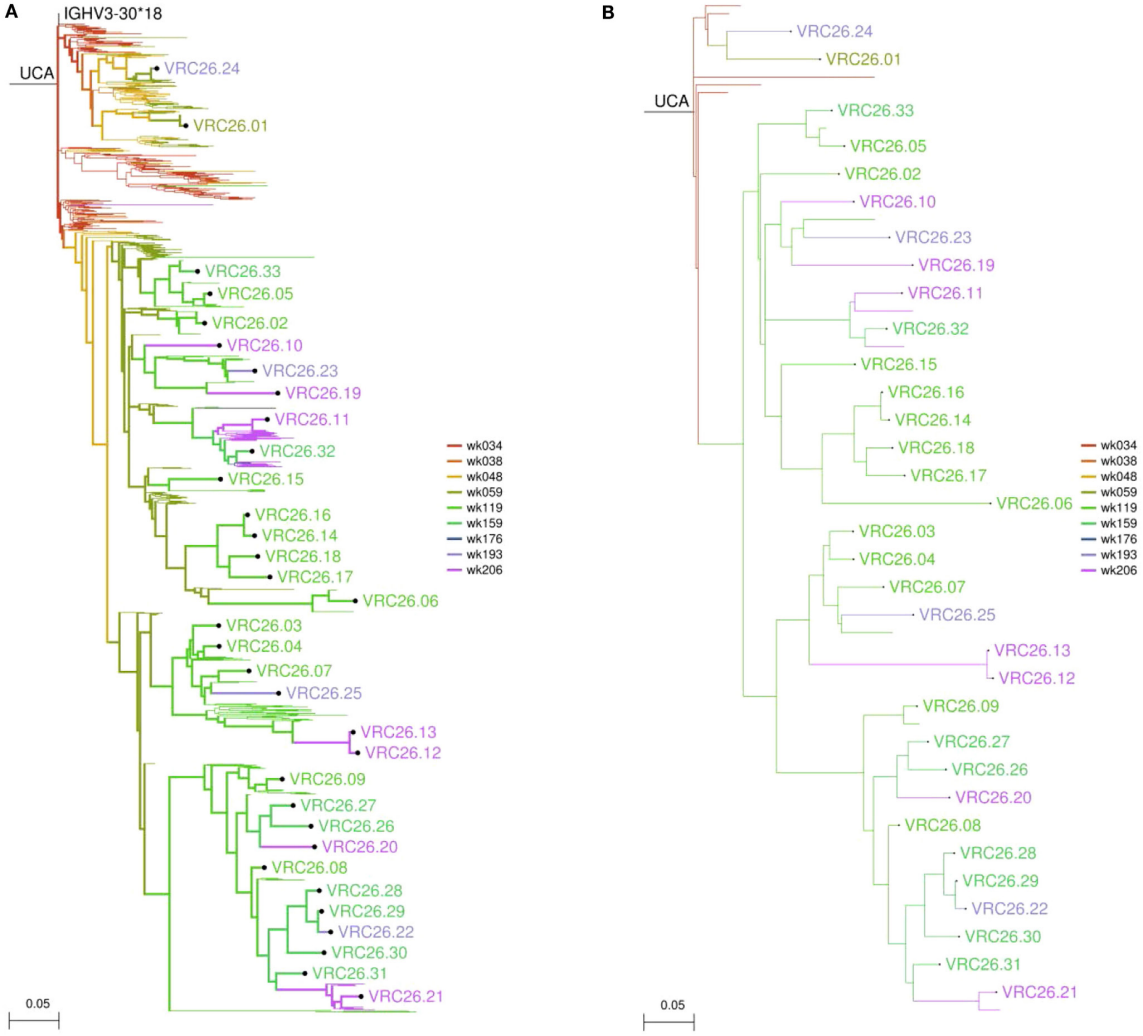
**Sample figures for Module 3 analyses**. **(A)** Longitudinal birthday tree of the CAP256-VRC26 lineage. This tree includes 384 NGS transcripts and the 33 isolated monoclonals. **(B)** The same tree displaying only 12 major branches derived from the NGS data and the 33 monoclonals. This allows the structure of the tree to be seen more clearly.

This is a wrapper script for using DNAML (47) to build a maximum likelihood tree representing the phylogenetic development of the lineage and to infer unobserved ancestral sequences. In most cases, the user should provide a manually verified, high-quality alignment in PHYLIP format, in order to allow for accurate inference of ancestor sequences. However, the program will call MUSCLE to align the collected transcripts if no alignment is provided. DNAML will be run three times on randomly ordered input, and outgroup rooted on the germline V gene sequence. All other options for DNAML are left at their default settings. The phylogenetic tree produced can be displayed using 4.4-display_tree.py (see below), and an example can be seen in Figure 4A.

#### 3.3-pick_intermediates.pl

This script analyzes the phylogenetic tree and ancestral sequences inferred by DNAML to pick developmental intermediates that show how a known antibody of interest evolved from the inferred unmutated common ancestor. The user may either specify how many approximately equally spaced intermediates should be selected or the approximate number of amino acid changes between consecutive intermediates. The script can also identify the inferred sequence for the most recent common ancestor of multiple antibodies of interest.

#### 3.4-collapse_minor_branches.pl

Often there are too many sequences (hundreds or thousands) to be clearly displayed on a phylogenetic tree. This script clusters lineage CDR3 sequences in a phylogenetically aware manner to produce a partially collapsed version of the phylogenetic tree emphasizing the major branches of the lineage. The identity threshold for clustering CDR3s and the minimum number of sequences required to define a “major” branch may be adjusted by the user. Known antibody sequences may be specified and will be displayed regardless of whether or not they are part of a major branch. The summary table will also indicate the temporal persistence of each major branch, where available. A collapsed version of the tree in Figure 4A is shown in Figure 4B.

#### 3.5-evolutionary_rate.pl

This script generates an xml-formatted configuration file for BEAST2 (48) to calculate the evolutionary rate of an antibody lineage. DNA sequences from at least two time points are required to run this script. The script can separate antibody variable region sequences into different partitions and generate configuration files to calculate the evolutionary rates spontaneously for V(D)J region, CDR regions, framework regions, and the first + second and third codon positions (2, 12).

### Module 4: Figures and Output

The final module of SONAR produces figures visualizing the results of the analyses conducted by the other three modules.

#### 4.1-setup_plots.pl and 4.2-plot_histograms.R

These scripts plot histograms or bar charts to show the distributions of many different repertoire properties, such as transcript lengths, germline gene usage, SHM levels, and CDR3 net charge, among others. These properties may be calculated for all transcripts in the raw data, all functional transcripts (successful V and J assignment, in-frame junction, and no stop codons), unique transcripts only (as determined by the parameters provided to 2.1-calulate_id-div.pl), or a manually specified subset of transcripts. Multiple repertoire features or data from multiple samples may be plotted on a single figure, as well, and many options are provided for adjusting the appearance of the final figure. All options are provided by the user to 4.1-setup_plots.pl, which extracts and reformats the required data and then automatically calls 4.2-plot_histograms.R to plot the data and generate the final figure. Sample plots are shown in Figure 2.

#### 4.3-plot_identity_divergence.R

This script uses the output of 2.1-calulate_id-div.pl to plot bulk NGS data as a heat map with the *x* axis corresponding to the divergence from the assigned germline V gene for each transcript and the *y* axis showing the full-length sequence identity to an antibody of interest. In these plots, transcripts from the same lineage as the antibody reference typically appear as clearly distinguishable islands separated from the main body of unrelated transcripts (11, 12) (Figure 3A). In addition, markers can be used to indicate the positions of specific transcripts, such as those identified by Module 2 as members of the same lineage (Figure 3B). Finally, multiple longitudinal datasets can be provided to generate a single figure with a row of identity–divergence plots showing the evolution of the repertoire over time.

#### 4.4-display_tree.py

This script uses the ete2 library (49) to generate publication-quality images of the trees output by 3.2-run_DNAML.py or 3.4-cluster_tree.pl. Each branch is colored by the birthday time point assigned by 3.1-merge_timepoints.pl. Options are provided to label both intermediates (internal nodes) and sequences (leaves/tips) of interest or to collapse specific branches of the tree. Additional options for adjusting various graphical parameters are also available. Sample trees are shown in Figure 4.

### Other Utility Scripts

A variety of additional stand-alone scripts are provided to help carry out common tasks. These include detecting frameshift mutations from pyrosequencing, subsetting sequence files, and manipulating phylogenetic trees in various ways.

### Data Vignette

We have previously used earlier versions of the SONAR scripts to analyze several lineages of broadly neutralizing antibodies targeting HIV-1, including the CAP256-VRC26 lineage (11, 39, 41). The raw sequencing data for donor CAP256 are available from the NCBI Sequence Reads Archive with accession number SRP034555. As a tutorial, SONAR includes the commands used to download these data and run the pipeline on it on the Docker container, along with the outputs produced.

## Discussion

Here, we present an integrated pipeline for analyzing NGS data of BCR transcripts to identify and to trace the development of a specific antibody lineage across multiple time points. This pipeline has already been used successfully to investigate multiple broadly neutralizing antibody lineages against HIV-1 (11, 12, 39, 41) and can easily be applied to other systems of interest, including antibodies against influenza virus and pathogenic autoantibodies.

Software for the Ontogenic aNalysis of Antibody Repertoires serves as an all-in-one solution, allowing a user to go from raw data to final analysis within a single ecosystem. With the recent proliferation of software for analyzing NGS data from BCR repertoires (55, 56), several specialized programs are available for assigning exact allelic origins and recombination points (27, 29). However, SONAR’s unique strength lies in the ability to easily identify transcripts related to an antibody of interest and, especially, to integrate sequences from multiple time points. Therefore, while SONAR assigns a particular allele to each transcript based on the BLAST output, all downstream analyses group the alleles of each germline gene in order to be more inclusive. SONAR is also explicitly agnostic as to the exact recombination points and P- and N-insertions within a specific antibody sequence. Importantly, because SONAR is focused on finding transcripts related to a known antibody, this imprecision can yield better results in the description of a lineage’s ontogeny. Moreover, by working with simple fasta-formatted sequence files, SONAR provides interoperability with these specialized tools, as well as with others devoted to dividing an entire repertoire into its component lineages [e.g., Ref. (57, see footnote text 1)].

Software for the Ontogenic aNalysis of Antibody Repertoires relies on a number of external programs and libraries, including BLAST+, MUSCLE, USEARCH, DNAML, and others. Because each of these may also have their own dependencies, setting up SONAR can be difficult. To increase the ease of use, we have created a fully setup Docker image,^4^ which can be downloaded and run on any computer or operating system without need for installation of any additional software.

The current version of SONAR closely resembles that used to carry out previously described analyses (2, 11, 12) and provides a fully functional, integrated pipeline for the ontogenic analysis of antibody repertories. In addition, SONAR remains under active development. Current focuses include a module to estimate functional selection pressure dynamics over time for antibody lineages (2). As we have shown that mutability and substitution bias modulate how somatic hypermutation occurs at each position in the antibody variable region (10), a module to characterize germline gene-specific mutational profiles from transcripts sampled by NGS would allow estimation of how likely certain mutation patterns are to be reproduced in either natural infection or vaccination. Other new functionalities are also being developed, and both bug fixes and new features will be added to the GitHub repository as they become available.

## Author Contributions

CS, ZS, ZZ, JM, PK, and LS designed the analyses to be included in the SONAR suite. CS and ZZ built SONAR’s underlying architecture. CS, ZS, and ZZ wrote the code. CS wrote the manuscript. All authors reviewed, commented on, and approved the manuscript.

## Conflict of Interest Statement

The authors declare that the research was conducted in the absence of any commercial or financial relationships that could be construed as a potential conflict of interest.

## Footnotes

^1^Ralph DK, Matsen FA. *Likelihood-Based Inference of B-Cell Clonal Families*. (2016). in press. Available from: https://arxiv.org/abs/1603.08127

^2^http://hannonlab.cshl.edu/fastx_toolkit/

^3^www.docopt.org

^4^https://hub.docker.com/r/scharch/sonar/
